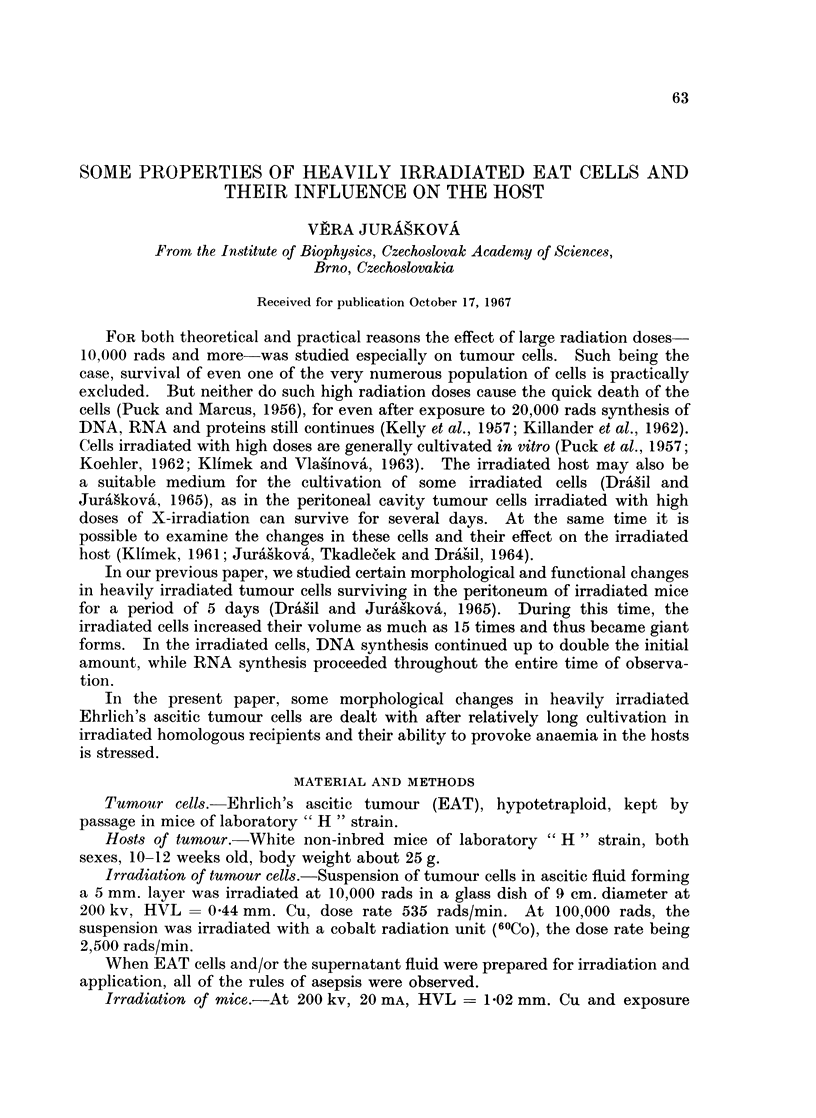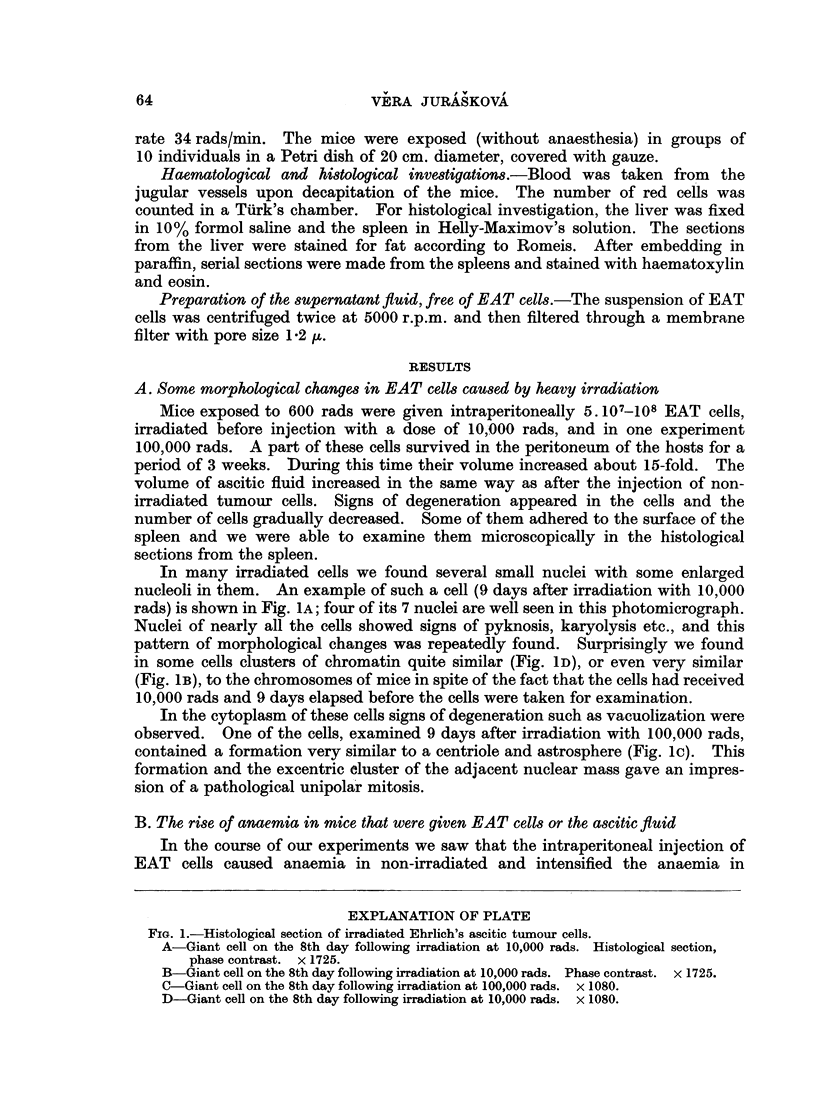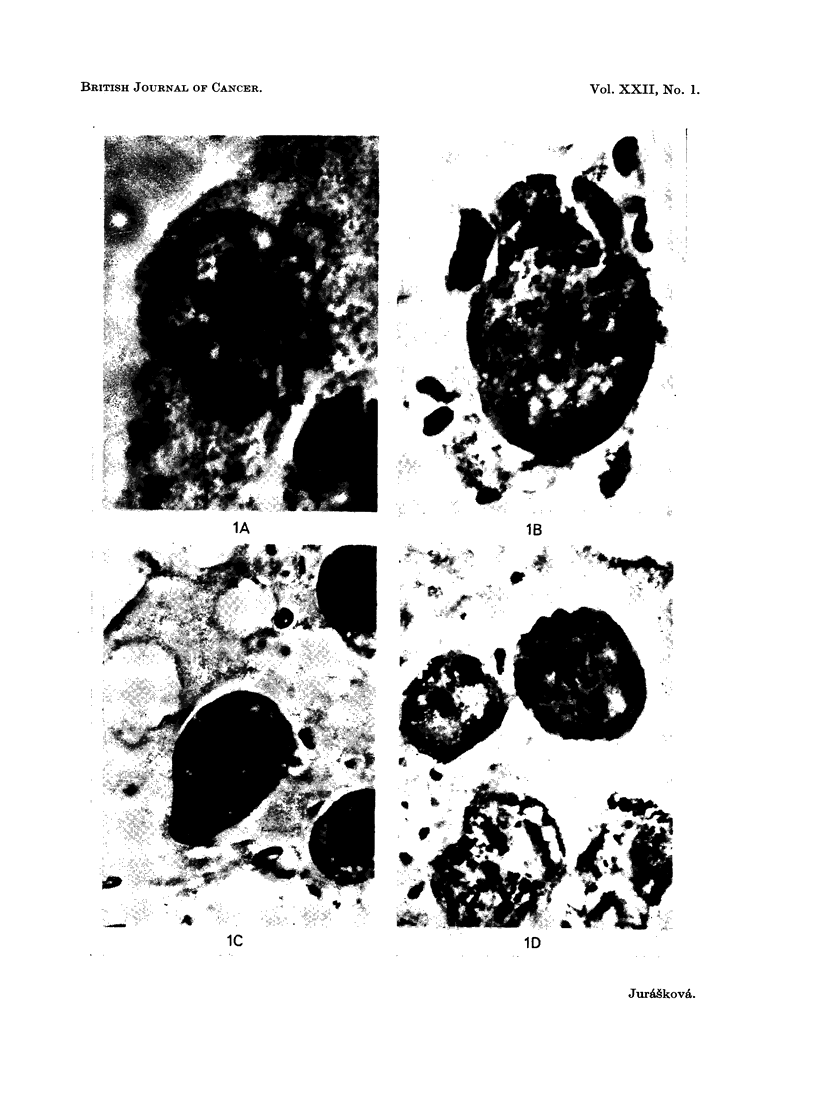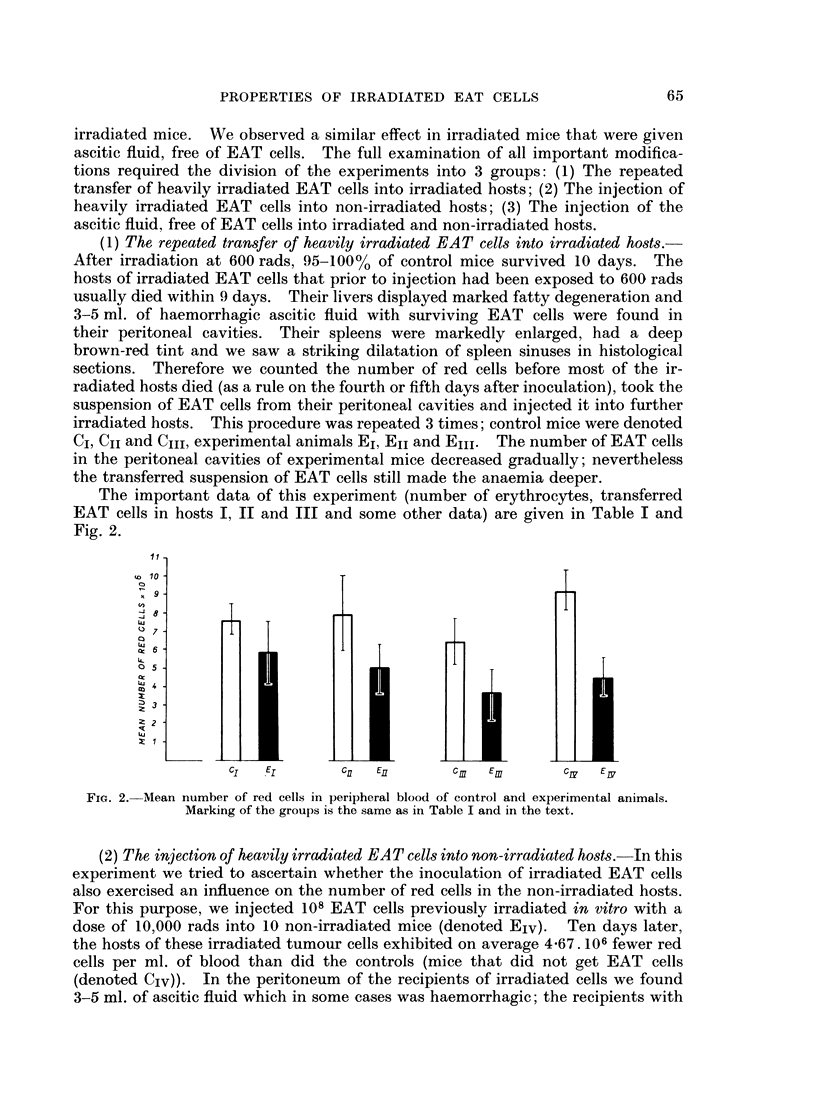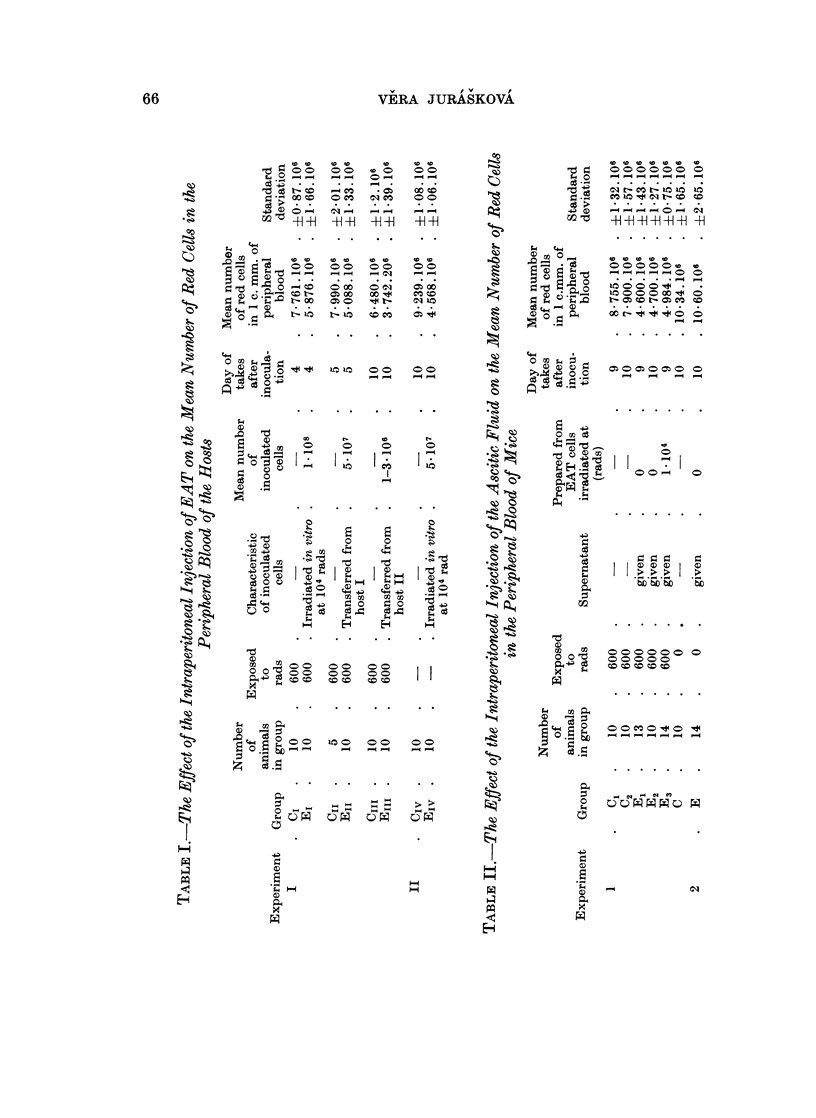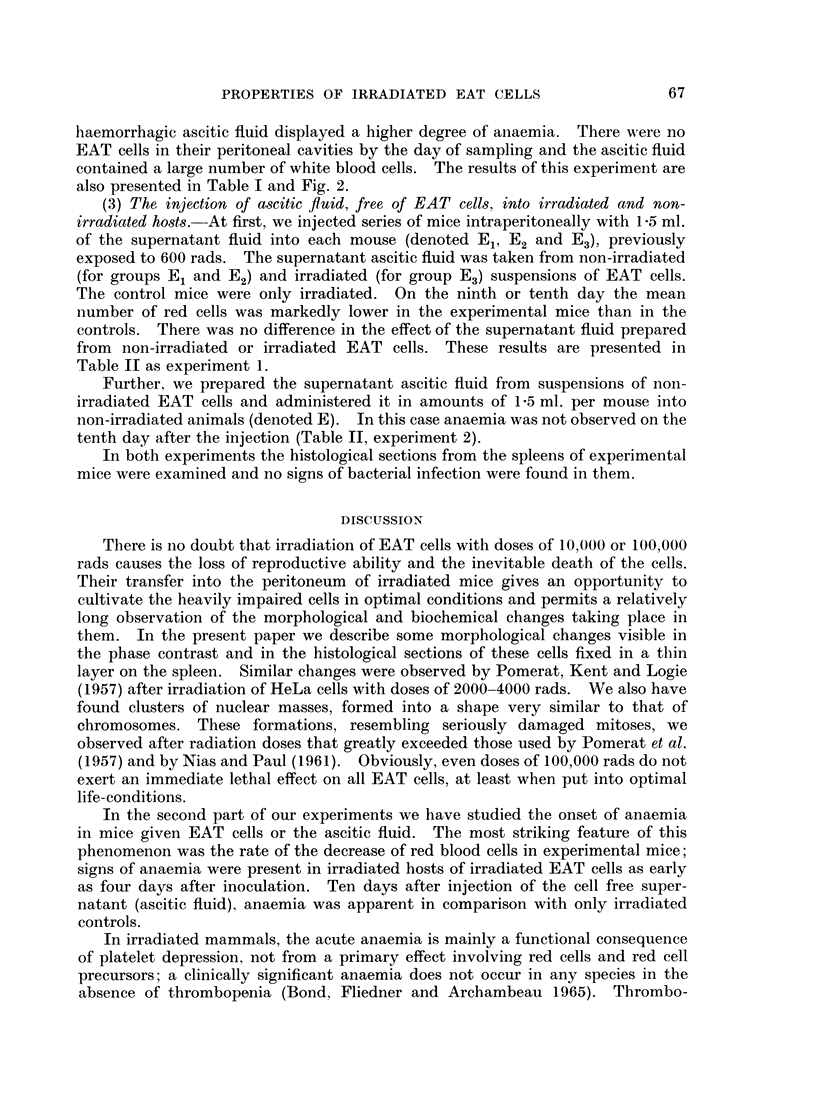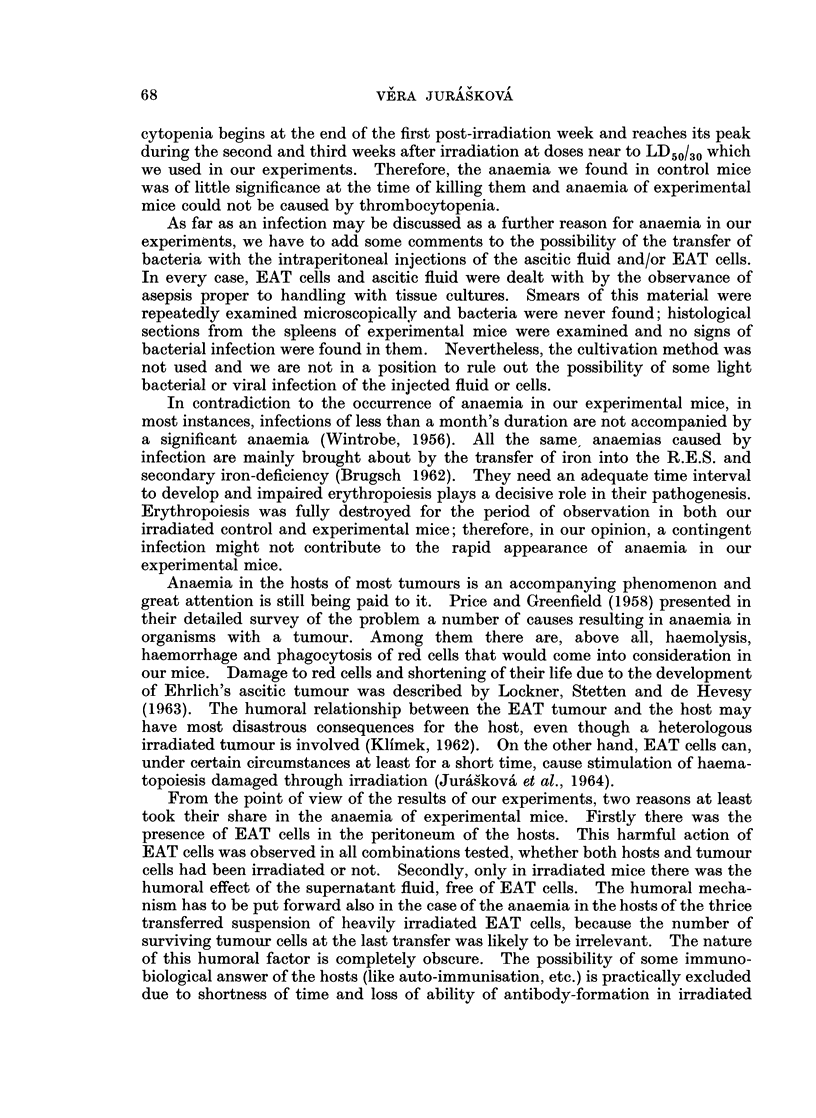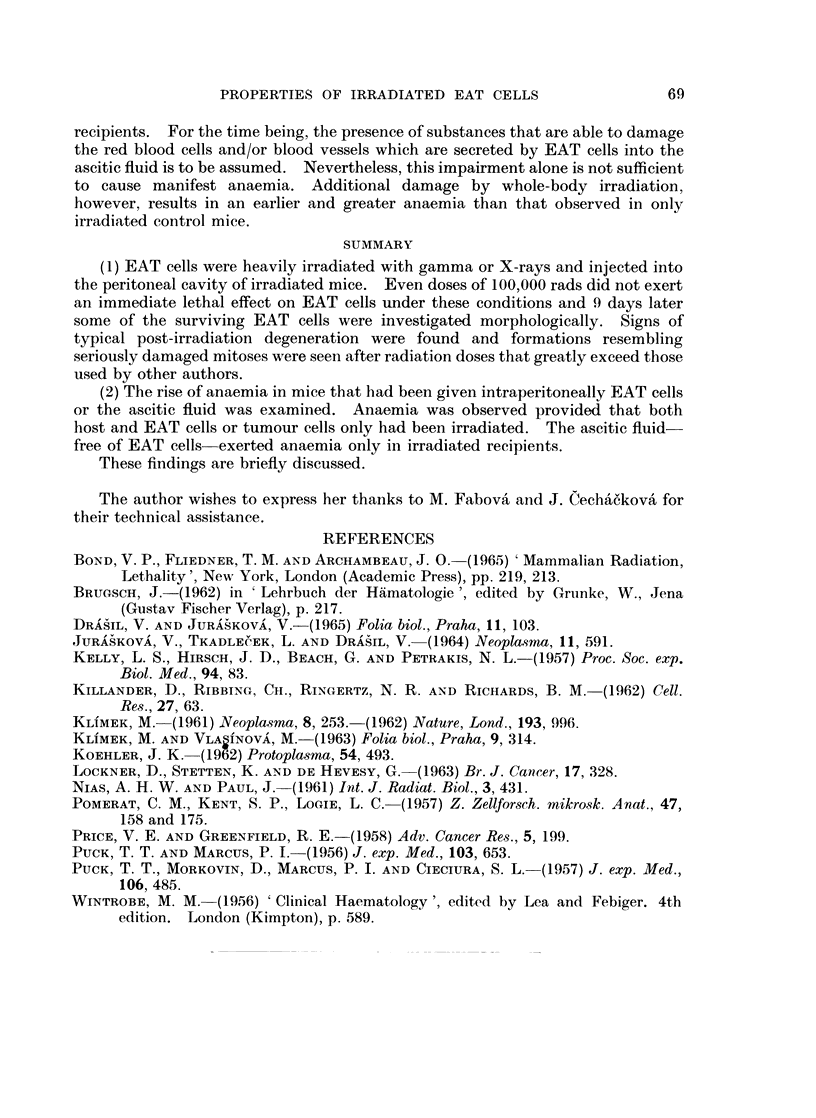# Some properties of heavily irradiated EAT cells and their influence on the host.

**DOI:** 10.1038/bjc.1968.9

**Published:** 1968-03

**Authors:** V. Jurásková

## Abstract

**Images:**


### SOME PROPERTIES OF HEAVILY IRRADIATED EAT CELLS AND THEIR INFLUENCE ON THE HOST

VĚRA JURÁŠKOVÁ

*From the Institute of Biophysics, Czechoslovak Academy of Sciences, Brno, Czechoslovakia*

Received for publication October 17, 1967

FOR both theoretical and practical reasons the effect of large radiation doses—10,000 rads and more—was studied especially on tumour cells. Such being the case, survival of even one of the very numerous population of cells is practically excluded. But neither do such high radiation doses cause the quick death of the cells (Puck and Marcus, 1956), for even after exposure to 20,000 rads synthesis of DNA, RNA and proteins still continues (Kelly *et al.*, 1957; Killander *et al.*, 1962). Cells irradiated with high doses are generally cultivated *in vitro* (Puck *et al.*, 1957; Koehler, 1962; Klímek and Vlašínová, 1963). The irradiated host may also be a suitable medium for the cultivation of some irradiated cells (Drášil and Jurášková, 1965), as in the peritoneal cavity tumour cells irradiated with high doses of X-irradiation can survive for several days. At the same time it is possible to examine the changes in these cells and their effect on the irradiated host (Klímek, 1961; Jurášková, Tkadleček and Drášil, 1964).

In our previous paper, we studied certain morphological and functional changes in heavily irradiated tumour cells surviving in the peritoneum of irradiated mice for a period of 5 days (Drášil and Jurášková, 1965). During this time, the irradiated cells increased their volume as much as 15 times and thus became giant forms. In the irradiated cells, DNA synthesis continued up to double the initial amount, while RNA synthesis proceeded throughout the entire time of observation.

In the present paper, some morphological changes in heavily irradiated Ehrlich's ascitic tumour cells are dealt with after relatively long cultivation in irradiated homologous recipients and their ability to provoke anaemia in the hosts is stressed.

### MATERIAL AND METHODS

*Tumour cells.*—Ehrlich's ascitic tumour (EAT), hypotetraploid, kept by passage in mice of laboratory " H " strain.

*Hosts of tumour.*—White non-inbred mice of laboratory " H " strain, both sexes, 10–12 weeks old, body weight about 25 g.

*Irradiation of tumour cells.*—Suspension of tumour cells in ascitic fluid forming a 5 mm. layer was irradiated at 10,000 rads in a glass dish of 9 cm. diameter at 200 kv, HVL = 0·44 mm. Cu, dose rate 535 rads/min. At 100,000 rads, the suspension was irradiated with a cobalt radiation unit ($^{60}Co$), the dose rate being 2,500 rads/min.

When EAT cells and/or the supernatant fluid were prepared for irradiation and application, all of the rules of asepsis were observed.

*Irradiation of mice.*—At 200 kv, 20 mA, HVL = 1·02 mm. Cu and exposure rate 34 rads/min. The mice were exposed (without anaesthesia) in groups of 10 individuals in a Petri dish of 20 cm. diameter, covered with gauze.

*Haematological and histological investigations.*—Blood was taken from the jugular vessels upon decapitation of the mice. The number of red cells was counted in a Türk's chamber. For histological investigation, the liver was fixed in 10% formol saline and the spleen in Helly-Maximov's solution. The sections from the liver were stained for fat according to Romeis. After embedding in paraffin, serial sections were made from the spleens and stained with haematoxylin and eosin.

*Preparation of the supernatant fluid, free of EAT cells.*—The suspension of EAT cells was centrifuged twice at 5000 r.p.m. and then filtered through a membrane filter with pore size 1·2 $\mu$.

### RESULTS

### *A. Some morphological changes in EAT cells caused by heavy irradiation*

Mice exposed to 600 rads were given intraperitoneally $5.10^7$–$10^8$ EAT cells, irradiated before injection with a dose of 10,000 rads, and in one experiment 100,000 rads. A part of these cells survived in the peritoneum of the hosts for a period of 3 weeks. During this time their volume increased about 15-fold. The volume of ascitic fluid increased in the same way as after the injection of non-irradiated tumour cells. Signs of degeneration appeared in the cells and the number of cells gradually decreased. Some of them adhered to the surface of the spleen and we were able to examine them microscopically in the histological sections from the spleen.

In many irradiated cells we found several small nuclei with some enlarged nucleoli in them. An example of such a cell (9 days after irradiation with 10,000 rads) is shown in Fig. 1A; four of its 7 nuclei are well seen in this photomicrograph. Nuclei of nearly all the cells showed signs of pyknosis, karyolysis etc., and this pattern of morphological changes was repeatedly found. Surprisingly we found in some cells clusters of chromatin quite similar (Fig. 1D), or even very similar (Fig. 1B), to the chromosomes of mice in spite of the fact that the cells had received 10,000 rads and 9 days elapsed before the cells were taken for examination.

In the cytoplasm of these cells signs of degeneration such as vacuolization were observed. One of the cells, examined 9 days after irradiation with 100,000 rads, contained a formation very similar to a centriole and astrosphere (Fig. 1C). This formation and the excentric cluster of the adjacent nuclear mass gave an impression of a pathological unipolar mitosis.

### B. *The rise of anaemia in mice that were given EAT cells or the ascitic fluid*

In the course of our experiments we saw that the intraperitoneal injection of EAT cells caused anaemia in non-irradiated and intensified the anaemia in

---

EXPLANATION OF PLATE

FIG. 1.—Histological section of irradiated Ehrlich's ascitic tumour cells.

A—Giant cell on the 8th day following irradiation at 10,000 rads. Histological section, phase contrast. × 1725.

B—Giant cell on the 8th day following irradiation at 10,000 rads. Phase contrast. × 1725.

C—Giant cell on the 8th day following irradiation at 100,000 rads. × 1080.

D—Giant cell on the 8th day following irradiation at 10,000 rads. × 1080.

1A 1B

1C 1D

Jurášková.

irradiated mice. We observed a similar effect in irradiated mice that were given ascitic fluid, free of EAT cells. The full examination of all important modifications required the division of the experiments into 3 groups: (1) The repeated transfer of heavily irradiated EAT cells into irradiated hosts; (2) The injection of heavily irradiated EAT cells into non-irradiated hosts; (3) The injection of the ascitic fluid, free of EAT cells into irradiated and non-irradiated hosts.

(1) *The repeated transfer of heavily irradiated EAT cells into irradiated hosts.*—After irradiation at 600 rads, 95–100% of control mice survived 10 days. The hosts of irradiated EAT cells that prior to injection had been exposed to 600 rads usually died within 9 days. Their livers displayed marked fatty degeneration and 3–5 ml. of haemorrhagic ascitic fluid with surviving EAT cells were found in their peritoneal cavities. Their spleens were markedly enlarged, had a deep brown-red tint and we saw a striking dilatation of spleen sinuses in histological sections. Therefore we counted the number of red cells before most of the irradiated hosts died (as a rule on the fourth or fifth days after inoculation), took the suspension of EAT cells from their peritoneal cavities and injected it into further irradiated hosts. This procedure was repeated 3 times; control mice were denoted $C_I$, $C_{II}$ and $C_{III}$, experimental animals $E_I$, $E_{II}$ and $E_{III}$. The number of EAT cells in the peritoneal cavities of experimental mice decreased gradually; nevertheless the transferred suspension of EAT cells still made the anaemia deeper.

The important data of this experiment (number of erythrocytes, transferred EAT cells in hosts I, II and III and some other data) are given in Table I and Fig. 2.

FIG. 2.—Mean number of red cells in peripheral blood of control and experimental animals. Marking of the groups is the same as in Table I and in the text.

(2) *The injection of heavily irradiated EAT cells into non-irradiated hosts.*—In this experiment we tried to ascertain whether the inoculation of irradiated EAT cells also exercised an influence on the number of red cells in the non-irradiated hosts. For this purpose, we injected $10^8$ EAT cells previously irradiated *in vitro* with a dose of 10,000 rads into 10 non-irradiated mice (denoted $E_{IV}$). Ten days later, the hosts of these irradiated tumour cells exhibited on average $4{\cdot}67 . 10^6$ fewer red cells per ml. of blood than did the controls (mice that did not get EAT cells (denoted $C_{IV}$)). In the peritoneum of the recipients of irradiated cells we found 3–5 ml. of ascitic fluid which in some cases was haemorrhagic; the recipients with

TABLE I.—*The Effect of the Intraperitoneal Injection of EAT on the Mean Number of Red Cells in the Peripheral Blood of the Hosts*

| Experiment | Group | Number of animals in group | Exposed to rads | Characteristic of inoculated cells | Mean number of inoculated cells | Day of takes after inoculation | Mean number of red cells in 1 c. mm. of peripheral blood | Standard deviation |
|---|---|---|---|---|---|---|---|---|
| I | $C_{I}$ | 10 | 600 | — | — | 4 | $7{\cdot}761.10^6$ | $\pm 0{\cdot}87.10^6$ |
| | $E_{I}$ | 10 | 600 | Irradiated *in vitro* at $10^4$ rads | $1{\cdot}10^8$ | 4 | $5{\cdot}876.10^6$ | $\pm 1{\cdot}66.10^6$ |
| | $C_{II}$ | 5 | 600 | — | — | 5 | $7{\cdot}990.10^6$ | $\pm 2{\cdot}01.10^6$ |
| | $E_{II}$ | 10 | 600 | Transferred from host I | $5{\cdot}10^7$ | 5 | $5{\cdot}088.10^6$ | $\pm 1{\cdot}33.10^6$ |
| | $C_{III}$ | 10 | 600 | — | — | 10 | $6{\cdot}480.10^6$ | $\pm 1{\cdot}2.10^6$ |
| | $E_{III}$ | 10 | 600 | Transferred from host II | $1\text{–}3{\cdot}10^6$ | 10 | $3{\cdot}742.20^6$ | $\pm 1{\cdot}39.10^6$ |
| II | $C_{IV}$ | 10 | — | — | — | 10 | $9{\cdot}239.10^6$ | $\pm 1{\cdot}08.10^6$ |
| | $E_{IV}$ | 10 | — | Irradiated *in vitro* at $10^4$ rad | $5{\cdot}10^7$ | 10 | $4{\cdot}568.10^6$ | $\pm 1{\cdot}06.10^6$ |

TABLE II.—*The Effect of the Intraperitoneal Injection of the Ascitic Fluid on the Mean Number of Red Cells in the Peripheral Blood of Mice*

| Experiment | Group | Number of animals in group | Exposed to rads | Supernatant | Prepared from EAT cells irradiated at (rads) | Day of takes after inoculation | Mean number of red cells in 1 c.mm. of peripheral blood | Standard deviation |
|---|---|---|---|---|---|---|---|---|
| 1 | $C_1$ | 10 | 600 | — | — | 9 | $8{\cdot}755.10^6$ | $\pm 1{\cdot}32.10^6$ |
| | $C_2$ | 10 | 600 | — | — | 10 | $7{\cdot}900.10^6$ | $\pm 1{\cdot}57.10^6$ |
| | $E_1$ | 13 | 600 | given | 0 | 9 | $4{\cdot}600.10^6$ | $\pm 1{\cdot}43.10^6$ |
| | $E_2$ | 10 | 600 | given | 0 | 10 | $4{\cdot}700.10^6$ | $\pm 1{\cdot}27.10^6$ |
| | $E_3$ | 14 | 600 | given | $1{\cdot}10^4$ | 9 | $4{\cdot}984.10^6$ | $\pm 0{\cdot}75.10^6$ |
| | C | 10 | 0 | — | — | 10 | $10{\cdot}34.10^6$ | $\pm 1{\cdot}65.10^6$ |
| 2 | E | 14 | 0 | given | 0 | 10 | $10{\cdot}60.10^6$ | $\pm 2{\cdot}65.10^6$ |

haemorrhagic ascitic fluid displayed a higher degree of anaemia. There were no EAT cells in their peritoneal cavities by the day of sampling and the ascitic fluid contained a large number of white blood cells. The results of this experiment are also presented in Table I and Fig. 2.

(3) *The injection of ascitic fluid, free of EAT cells, into irradiated and non-irradiated hosts.*—At first, we injected series of mice intraperitoneally with 1·5 ml. of the supernatant fluid into each mouse (denoted $E_1$, $E_2$ and $E_3$), previously exposed to 600 rads. The supernatant ascitic fluid was taken from non-irradiated (for groups $E_1$ and $E_2$) and irradiated (for group $E_3$) suspensions of EAT cells. The control mice were only irradiated. On the ninth or tenth day the mean number of red cells was markedly lower in the experimental mice than in the controls. There was no difference in the effect of the supernatant fluid prepared from non-irradiated or irradiated EAT cells. These results are presented in Table II as experiment 1.

Further, we prepared the supernatant ascitic fluid from suspensions of non-irradiated EAT cells and administered it in amounts of 1·5 ml. per mouse into non-irradiated animals (denoted E). In this case anaemia was not observed on the tenth day after the injection (Table II, experiment 2).

In both experiments the histological sections from the spleens of experimental mice were examined and no signs of bacterial infection were found in them.

### DISCUSSION

There is no doubt that irradiation of EAT cells with doses of 10,000 or 100,000 rads causes the loss of reproductive ability and the inevitable death of the cells. Their transfer into the peritoneum of irradiated mice gives an opportunity to cultivate the heavily impaired cells in optimal conditions and permits a relatively long observation of the morphological and biochemical changes taking place in them. In the present paper we describe some morphological changes visible in the phase contrast and in the histological sections of these cells fixed in a thin layer on the spleen. Similar changes were observed by Pomerat, Kent and Logie (1957) after irradiation of HeLa cells with doses of 2000–4000 rads. We also have found clusters of nuclear masses, formed into a shape very similar to that of chromosomes. These formations, resembling seriously damaged mitoses, we observed after radiation doses that greatly exceeded those used by Pomerat *et al.* (1957) and by Nias and Paul (1961). Obviously, even doses of 100,000 rads do not exert an immediate lethal effect on all EAT cells, at least when put into optimal life-conditions.

In the second part of our experiments we have studied the onset of anaemia in mice given EAT cells or the ascitic fluid. The most striking feature of this phenomenon was the rate of the decrease of red blood cells in experimental mice; signs of anaemia were present in irradiated hosts of irradiated EAT cells as early as four days after inoculation. Ten days after injection of the cell free supernatant (ascitic fluid), anaemia was apparent in comparison with only irradiated controls.

In irradiated mammals, the acute anaemia is mainly a functional consequence of platelet depression, not from a primary effect involving red cells and red cell precursors; a clinically significant anaemia does not occur in any species in the absence of thrombopenia (Bond, Fliedner and Archambeau 1965). Thrombocytopenia begins at the end of the first post-irradiation week and reaches its peak during the second and third weeks after irradiation at doses near to $LD_{50/30}$ which we used in our experiments. Therefore, the anaemia we found in control mice was of little significance at the time of killing them and anaemia of experimental mice could not be caused by thrombocytopenia.

As far as an infection may be discussed as a further reason for anaemia in our experiments, we have to add some comments to the possibility of the transfer of bacteria with the intraperitoneal injections of the ascitic fluid and/or EAT cells. In every case, EAT cells and ascitic fluid were dealt with by the observance of asepsis proper to handling with tissue cultures. Smears of this material were repeatedly examined microscopically and bacteria were never found; histological sections from the spleens of experimental mice were examined and no signs of bacterial infection were found in them. Nevertheless, the cultivation method was not used and we are not in a position to rule out the possibility of some light bacterial or viral infection of the injected fluid or cells.

In contradiction to the occurrence of anaemia in our experimental mice, in most instances, infections of less than a month's duration are not accompanied by a significant anaemia (Wintrobe, 1956). All the same, anaemias caused by infection are mainly brought about by the transfer of iron into the R.E.S. and secondary iron-deficiency (Brugsch 1962). They need an adequate time interval to develop and impaired erythropoiesis plays a decisive role in their pathogenesis. Erythropoiesis was fully destroyed for the period of observation in both our irradiated control and experimental mice; therefore, in our opinion, a contingent infection might not contribute to the rapid appearance of anaemia in our experimental mice.

Anaemia in the hosts of most tumours is an accompanying phenomenon and great attention is still being paid to it. Price and Greenfield (1958) presented in their detailed survey of the problem a number of causes resulting in anaemia in organisms with a tumour. Among them there are, above all, haemolysis, haemorrhage and phagocytosis of red cells that would come into consideration in our mice. Damage to red cells and shortening of their life due to the development of Ehrlich's ascitic tumour was described by Lockner, Stetten and de Hevesy (1963). The humoral relationship between the EAT tumour and the host may have most disastrous consequences for the host, even though a heterologous irradiated tumour is involved (Klímek, 1962). On the other hand, EAT cells can, under certain circumstances at least for a short time, cause stimulation of haematopoiesis damaged through irradiation (Jurášková *et al.*, 1964).

From the point of view of the results of our experiments, two reasons at least took their share in the anaemia of experimental mice. Firstly there was the presence of EAT cells in the peritoneum of the hosts. This harmful action of EAT cells was observed in all combinations tested, whether both hosts and tumour cells had been irradiated or not. Secondly, only in irradiated mice there was the humoral effect of the supernatant fluid, free of EAT cells. The humoral mechanism has to be put forward also in the case of the anaemia in the hosts of the thrice transferred suspension of heavily irradiated EAT cells, because the number of surviving tumour cells at the last transfer was likely to be irrelevant. The nature of this humoral factor is completely obscure. The possibility of some immunobiological answer of the hosts (like auto-immunisation, etc.) is practically excluded due to shortness of time and loss of ability of antibody-formation in irradiated recipients. For the time being, the presence of substances that are able to damage the red blood cells and/or blood vessels which are secreted by EAT cells into the ascitic fluid is to be assumed. Nevertheless, this impairment alone is not sufficient to cause manifest anaemia. Additional damage by whole-body irradiation, however, results in an earlier and greater anaemia than that observed in only irradiated control mice.

### SUMMARY

(1) EAT cells were heavily irradiated with gamma or X-rays and injected into the peritoneal cavity of irradiated mice. Even doses of 100,000 rads did not exert an immediate lethal effect on EAT cells under these conditions and 9 days later some of the surviving EAT cells were investigated morphologically. Signs of typical post-irradiation degeneration were found and formations resembling seriously damaged mitoses were seen after radiation doses that greatly exceed those used by other authors.

(2) The rise of anaemia in mice that had been given intraperitoneally EAT cells or the ascitic fluid was examined. Anaemia was observed provided that both host and EAT cells or tumour cells only had been irradiated. The ascitic fluid—free of EAT cells—exerted anaemia only in irradiated recipients.

These findings are briefly discussed.

The author wishes to express her thanks to M. Fabová and J. Čecháčková for their technical assistance.

### REFERENCES

Bond, V. P., Fliedner, T. M. and Archambeau, J. O.—(1965) ' Mammalian Radiation, Lethality ', New York, London (Academic Press), pp. 219, 213.

Brugsch, J.—(1962) in ' Lehrbuch der Hämatologie ', edited by Grunke, W., Jena (Gustav Fischer Verlag), p. 217.

Drášil, V. and Jurášková, V.—(1965) *Folia biol., Praha*, **11**, 103.

Jurášková, V., Tkadleček, L. and Drášil, V.—(1964) *Neoplasma*, **11**, 591.

Kelly, L. S., Hirsch, J. D., Beach, G. and Petrakis, N. L.—(1957) *Proc. Soc. exp. Biol. Med.*, **94**, 83.

Killander, D., Ribbing, Ch., Ringertz, N. R. and Richards, B. M.—(1962) *Cell. Res.*, **27**, 63.

Klímek, M.—(1961) *Neoplasma*, **8**, 253.—(1962) *Nature, Lond.*, **193**, 996.

Klímek, M. and Vlašínová, M.—(1963) *Folia biol., Praha*, **9**, 314.

Koehler, J. K.—(1962) *Protoplasma*, **54**, 493.

Lockner, D., Stetten, K. and de Hevesy, G.—(1963) *Br. J. Cancer*, **17**, 328.

Nias, A. H. W. and Paul, J.—(1961) *Int. J. Radiat. Biol.*, **3**, 431.

Pomerat, C. M., Kent, S. P., Logie, L. C.—(1957) *Z. Zellforsch. mikrosk. Anat.*, **47**, 158 and 175.

Price, V. E. and Greenfield, R. E.—(1958) *Adv. Cancer Res.*, **5**, 199.

Puck, T. T. and Marcus, P. I.—(1956) *J. exp. Med.*, **103**, 653.

Puck, T. T., Morkovin, D., Marcus, P. I. and Cieciura, S. L.—(1957) *J. exp. Med.*, **106**, 485.

Wintrobe, M. M.—(1956) ' Clinical Haematology ', edited by Lea and Febiger. 4th edition. London (Kimpton), p. 589.